# Global event-based surveillance of chemical incidents

**DOI:** 10.1038/s41370-021-00384-8

**Published:** 2021-11-08

**Authors:** Tom Gaulton, Charlotte Hague, David Cole, Eirian Thomas, Raquel Duarte-Davidson

**Affiliations:** Centre for Radiation, Chemical and Environmental Hazards, Public Health England, London, UK

**Keywords:** Chemicals, Chemical exposure, Chemical incident, Surveillance, EBS

## Abstract

**Background:**

The number of chemicals in our society and in our daily lives continues to increase. Accompanying this is an increasing risk of human exposure to and injury from hazardous substances. Performing regular, structured surveillance of chemical incidents allows a greater awareness of the types of chemical hazards causing injury and the frequency of their occurrence, as well as providing a better understanding of exposures.

**Objective:**

The objective of performing event-based surveillance (EBS) and capturing chemical incidents is to use this information to increase the situational awareness of chemical incidents, improve the management of these incidents and to inform measures to protect public health.

**Methods:**

This paper describes a method for EBS for chemical incidents, including the sources used, storing the gathered information and subsequent analysis of potential trends in the data.

**Results:**

We describe trends in the type of incidents that have been detected, the chemicals involved in these incidents and the health effects caused, in different geographic regions of the world.

**Significance:**

The methodology presented here provides a rapid and simple means of identifying chemical incidents that can be set up rapidly and with minimal cost, the outputs of which can be used to identify emerging risks and inform preparedness planning, response and training for chemical incidents.

## Introduction

Given the ever-increasing number and variety of chemicals manufactured, transported and used globally in all aspects of modern life [1], the risk of human exposures to these chemicals and subsequent health effects also increases. The World Health Organisation (WHO) defines a chemical incident as ‘the uncontrolled release of a toxic substance, potentially resulting in harm to public health and the environment’ [2]. These chemical incidents may be caused by industrial and domestic accidents or natural disasters, in addition to deliberate releases (e.g., due to terrorist activities). The chemicals involved in these incidents can include medicines, food additives, toxic industrial chemicals, illicit drugs and domestic chemicals (e.g., cleaning products). These agents may have immediate acute effects, and/or chronic long-term effects, depending on such factors as the dose of the chemical and the duration and route of exposure (e.g., dermal/eye absorption, ingestion and inhalation) [3]. Chemical incidents can be acute (e.g., industrial explosions) or longer-term incidents (such as prolonged exposure to environmental pollutants). They can also be localised, such as a release from a storage vessel, or widespread, such as a contaminated consumer product [4]. The impacts of chemical incidents can also occur very rapidly when compared to other hazards such as infectious diseases and as such, requires a much faster response to treat exposed individuals and prevent further injury [5].

In order to strengthen preparedness for chemical incidents, surveillance is a key activity required under the International Health Regulations core capacities (IHR, 2005) and the EU decision on cross-border health threats (Dec 1082/2013/EU – currently under review) [6, 7]. As outlined in the IHR (2005), ‘surveillance’ refers to the systematic ongoing collection, collation and analysis of data for public health purposes and the timely dissemination of public health information for assessment and public health response as necessary [6].

Event-based surveillance (EBS) is a functional component of the early warning and response process and encompasses the organised collection, monitoring, assessment and interpretation of mainly unstructured information (from formal and informal sources, e.g., official news websites and social media) regarding chemical incidents or hazards, which may represent an acute risk to human health [2]. EBS can be used to heighten situational awareness for current chemical incidents occurring globally (i.e., types of agents involved and the level of morbidity/mortality they cause). While this methodology is most commonly used for surveillance of infectious disease outbreaks [8–10], it is also useful for the monitoring of chemical incidents, such as poisoning incidents, explosions and fires, water/food contamination and also environmental incidents that present a hazard to public health, as many of the same reporting sources are used for both biological and chemical surveillance [11].

The benefits of using EBS for the surveillance, reporting and responding to chemical threats include its speed and cost-effectiveness, as few resources are required to perform EBS and ease of access to information (e.g., scanning social media) can sometimes provide more information from those on the ground than in official reports on the incident. More credible media sources usually provide accurate information and these are readily accessible via the internet [12]. The use of social media and internet news sites may also provide real-time data on chemical and other public health incidents with a level of detail not achievable using more traditional surveillance systems (e.g., indicator-based surveillance, syndromic surveillance or sentinel surveillance) [3]. This includes chemical incidents that occur in countries, which may not have appropriate surveillance and reporting systems, resources and experienced public health professionals.

However, there are some obvious caveats to this approach, the reports captured from websites may often not be verifiable, may include opinions or observations rather than established facts and may change over time as the incident unfolds, e.g., number of injuries and deaths. Sometimes when an incident is reported, the information does not include the identity of the chemical agent involved. Particularly when an incident occurs in countries with fewer public health resources and reduced analytical laboratory capacity, the identity is often not elucidated at all. Incidents may be reported for being newsworthy rather than an accurate depiction of its risk or impact to public health, e.g., when an explosion or release occurs but actually poses little risk to the public. In addition, when the media in a country is restricted or controlled, conflicting reports of the incident can arise (such as different chemicals being reported) or may not be reported at all. Despite these caveats, the rapidity at which this information is available allows the alerting of other partners/agencies and drafting of an initial risk assessment of the incident. This latter point is important when considering cross-border chemical health threats, as an initial risk assessment provides public health organisations in other countries with an early warning so that measures can be put into place earlier to limit the threat to public health [3].

EBS heightens the awareness of the individuals and organisations performing surveillance, this information could assist in identifying trends in chemical incidents and go on to inform public health interventions. Sharing this information with colleagues, collaborators and other stakeholders (in different organisations and countries) could lead to strengthening of preparedness and response activities nationally and internationally [13]; in addition, the recorded events can add value to other activities such as training and exercising staff on chemical incidents, e.g., through case studies, workshop activities, desktop exercises and fictional scenarios.

This paper describes the EBS methodology led by Public Health England that has been developed over a number of years through various international projects and involved a number of people in an international network from different organisations. The EBS strategy was originally initiated to detect chemical incidents within the EU or with the potential to affect EU citizens. Once detected, these could be posted to the EU Rapid Alerting System for CHEMicals (RASCHEM) for information sharing and awareness raising for incidents that may cross borders, under the EU ECHEMNET project [14]. The scope was then expanded to cover the rest of the globe and the incidents were used to inform global public health work and raise awareness of the detected incidents amongst colleagues, in addition to a notification and alerting mechanism.

EBS has contributed to surveillance activities and supports provisions within EU Decision 1082/2013 and IHR (2005). EBS also contributes to improving the process of detecting, analysing and notifying others of serious chemical health threats, including those that have the potential to cross international borders, which require a joined-up approach from multiple sectors and countries.

## Methods

### EBS strategy

In 2014, the initial EBS strategy was undertaken to capture incidents that were cross-border (or had the potential to be cross-border) in nature within the EU; however, the strategy was also able to detect chemical incidents from around the globe and those were also recorded in the database. Incidents that meet the criteria outlined in the methods are logged in a Microsoft Access database (see below for details). The websites used to perform EBS searches are detailed in Table 1. Many of them are used primarily for communicable disease surveillance, but also contain examples of chemical incidents/exposures, while some are non-specific, e.g., Twitter. This in itself highlights some of the difficulties of performing chemical EBS, as there are very few sources dedicated to chemical incidents. These websites were checked three times per week using the search terms (Table 1).

**Table 1:** Websites used in the EBS strategy.

Website	Link	Brief description
BBC News	https://www.bbc.co.uk/news	Search terms include: ‘chemical’, ‘toxic’, ‘poison’ and ‘explosion’
MediSYS	https://medisys.newsbrief.eu/medisys/categoryedition/symptoms/en/chemical.html	Also check the following pages: ‘chemical accident’, chemical threat’ and ‘toxic’
ProMedMail	https://promedmail.org/	Search terms include: ‘chemical’, ‘toxic’ and ‘poison’
RSOE EDIS	http://hisz.rsoe.hu/alertmap/index2.php?area=eu	Check map for all markers of HAZMAT, explosion, fires and CBRN incidents
HealthMap	https://www.healthmap.org/en/	Search using terms: ‘poisoning’ and ‘environmental’
GPHIN	https://gphin.canada.ca/requires registration for access	Search globally for events: within 48 h; involving ‘Environmental’, ‘Chemical’, ‘Product’ and ‘substance abuse’ categories; and are in English.
InformationAware	http://www.informationaware.com/special-project/search	Search for events involving: ‘Chemical spill’, ‘Factory explosion’ and ‘Industrial explosion’
Twitter	https://twitter.com/search-advanced?lang=en-gb	Search terms: ‘chemical’, ‘toxic’ and ‘poison’
Google News	https://www.google.co.uk	Perform Google search for ‘chemical’, ‘toxic’ and ‘poison’, then select ‘News’ tab. Limit results to the past week
Google Alerts	https://www.google.co.uk/alerts	Create automated email updates using the search terms: ‘chemical’, ‘toxic’ and ‘poison’

### Criteria for recording and notification of chemical incidents

EBS captures the impact of chemical incidents on public health, including chemical exposures, releases, explosions and poisonings. The strategy focuses on incidents that result in injury or death, rather than incidents that result in near misses, property damage or environmental pollution, i.e., the focus is on the effects of these incidents to public health, rather than risks that do not result in injury (no impact on public health and therefore not in the scope of ECHEMNET). It should be noted that this method did not take into account mental health, which can also be affected by exposure to chemical incidents and can occur even if physical injury was not sustained. However, the lengthy follow-up required for diagnosis and treatment of mental health issues is out of the scope of EBS. For this database, injuries were recorded when someone was exposed to a chemical. All chemical incidents resulting in injury or death are collected and distributed to colleagues and stakeholders within PHE, from around Europe and globally. The collected incidents form a valuable resource of past incidents for lessons learned and for monitoring that area or type of incident for similar occurrences.

Based on the criteria below, incidents detected with the websites listed in Table 1 were collected and recorded (if points 1 and either 2 or 3 below are met) and used for notifying stakeholders of the incidents (if points 4 or 5 below are met):

1. An unexpected chemical incident including a release, explosion, contamination, fire involving chemicals
2. Any incident involving chemicals that caused mortality
3. Any incident involving chemicals that caused significant injury (e.g., multiple casualties)
4. Cross-border determination; is the incident near rivers/ports, near borders?
5. Any incident involving chemicals that may ‘spread’—leading to additional cases of mortality or injury

Those who would be notified, depending on the incident type/chemical agent/incident location, include PHE staff involved in responding to chemical incidents in the UK, interested individuals from European Poison Centres and public health agencies, WHO International/WHO Europe staff involved in chemical incident response and the EU Scientific Committee for Health, Environment and Emerging Risks (SCHEER) that coordinates response to cross-border chemical incidents in the EU. These stakeholders are notified in case a chemical incident is of interest, may affect their country (or has a potential to affect their country) or they may have previous experience of such an event which would be valuable to share.

### EBS database

While initially the detected incidents were listed in an Excel spreadsheet, the increasing numbers of incidents required a more robust system to cope with the amounts of data stored. As such, a Microsoft (MS) Access database was written for Office 365. Initially, the data were stored in MS Access but now exists as SharePoint Lists to enhance performance, with Access remaining as the user interface. The design of the database was straightforward and consists of four main entities: Incidents, Agents, References and Categories.

An incident can also be tagged with multiple categories, which help describe the incident. Detected incidents are copied into emails that are distributed internally for review after every search (three times per week), to provide an opportunity to discuss the incidents to see if notification or follow-up is required. These emails are imported, from MS Outlook, into a database table where the individual stories are then separated and parsed. Longitude and latitude of the location of the chemical incident are also recorded, allowing the creation of maps to show the global distribution of the detected incidents, their health effects and types of chemical agent.

## Results

The recorded incidents presented in these results relate to the period between November 2014 and June 2020, covering the global EBS strategy. As of the end of June 2020, the database contains 1592 recorded chemical incidents in 121 countries, involving 252 unique chemical agents. The original purpose of performing EBS was to identify incidents that had the potential to be cross-border in nature, some of these incidents were then forwarded to RASCHEM and to colleagues from around Europe within the ECHEMNET network [15]. These incidents included an outbreak of methanol poisoning in Turkey in October 2015 due to adulterated spirits [16], this was posted as there was a potential for this spirit to be sold in the EU; in July 2016, there was an incorrect formulation of a vitamin D supplement marketed to babies and young children in Denmark, the higher than stated levels of vitamin D led to 25 babies becoming sick [17] and was posted to RASCHEM as the product was available online; in August 2017, around 150 beachgoers were exposed to a chemical mist that drifted from the English channel onto Birling Gap beach on the south coast of England [18], the origin of the mist was unclear and the event posted to RASCHEM for information for European colleague and the potential for the mist to affect French coastlines.

As well as sharing these incidents with RASCHEM, EU colleagues, WHO and the European Monitoring Centre for Drugs and Drug Addiction, the collected incidents provided a useful source of information about the types of incidents which occur, their location, the agents involved and some of the health effects caused.

The top 5 countries with the highest number of incidents detected, comprising over half of the detected incidents, include USA, with 322 (20.2%), India with 225 (14.1%), UK with 130 (8.2%), China with 117 (7.3%) and Russia with 55 (3.5%) detected incidents, respectively. These figures may not necessarily represent countries where the highest number of incidents is occurring; it could be that some countries have more advanced reporting systems than others. This can lead to all incidents being reported in some countries, whereas only the most serious ones being widely reported in other countries. For example, the incidents detected in the USA are associated with 117 deaths and 6497 injuries, while those detected in India are associated with 989 deaths and 6749 injuries. The number of injuries is comparable, while the number of deaths is far higher in India. While this is not a rigorous analysis, it shows that chemical incidents detected in India give rise to more deaths than in the USA.

### Sources of EBS data

To highlight where the different incidents are detected, the incidents detected from the last 12 months have been compiled along with their source website. Table 2 shows the number of chemical incidents detected from each of the source websites from the last 12 months. The ‘Other’ entry includes articles where the source was not recorded, or that were picked up but not through searching the source websites directly, e.g., an incident may be found on a news website while looking for a different incident, which was detected through the source websites. This does highlight a limitation in that it can be difficult to establish the primary source of an incident (i.e., where it was first reported and by whom). Some incidents can be detected through multiple sources and there is no way of knowing with certainty what the original source is; however, for our purposes of detecting and alerting others of these incidents, it is not necessary to know this.

**Table 2:** Number of chemical incident articles detected from each source website from the last 12 months.

Source of article/report	Number of detected incidents
RSOE EDIS	68 (20.0%)
InformationAware	63 (18.5%)
Google Alerts	48 (14.1%)
GPHIN	36 (10.6%)
Google News	23 (6.8%)
BBC News	15 (4.4%)
HealthMap	13 (3.8%)
ProMedMail	10 (2.9%)
MediSYS	10 (2.9%)
Twitter	8 (2.4%)
Other	46 (13.5%)

While 45% of the detected incidents have come from two websites (RSOE EDIS and InformationAware), the spread of all the incidents shows the value of having a variety of sources, as many more incidents would be missed with a reduced number of sources. Using just one of the source websites would preclude the detection of many other relevant chemical incidents. There may be other reasons for this distribution between sources, e.g., the two most common sources, RSOE EDIS and InformationAware, are map-based and allow incidents to be picked up by eye much easier than GPHIN, e.g., which requires reading through hundreds of summaries to find relevant chemical incidents. There are some regions (e.g., South and Central America) that are consistently underrepresented in the chemical incidents detected, this could be due to a number of reasons, for instance, a general lack of reporting in the region(s), an under-reporting of less serious incidents or the incidents not being reported in English and hence not picked up by the sites used by EBS.

### Most common chemicals in the detected incidents

From the detected incidents, around two-thirds of these incidents gave the identity of the chemical involved. Of these, the most commonly identified chemicals are chlorine, involved in 105 detected incidents (6.6%), ammonia, involved in 89 incidents (5.6%), methanol, involved in 86 incidents (5.4%) and carbon monoxide, involved in 47 incidents (3.0%). Often when incidents are detected, the chemical agent is actually unknown and this accounts for 525 of these incidents (33.0% of the total number of recorded incidents). This may be explained by the reporting of incidents soon after they have occurred and before the agent is known, and frequently the unknown agent is not elucidated. However, while the exact agent is sometimes unidentified, the type of agent is usually given, e.g., the incident may involve an unknown gas, fuel or pesticide.

### Overview of health effects caused by the detected chemical incidents

To give an overview of the detected chemical incidents, Table 3 lists the recorded incidents organised by incident category, as the full list of individual agents is too large to display, along with the number of deaths and injuries recorded from the incidents under that category. The table shows that the most frequently occurring incident types do not necessarily cause the most health effects. Industrial accidents is the category with the highest number of detected incidents; however, Poison in food and drink has less than half of the number of detected incidents, yet causes many more deaths and injuries than industrial accidents. This demonstrates the range of incident types and the need to have adequate appropriate arrangements to prevent, detect and respond to chemical incidents in line with IHR and EU Decision 1082.

**Table 3:** List of incident categories with associated health effects.

Description	Count	No. of deaths	No. of injuries
Industrial accident (e.g., release of chemicals at a manufacturing facility)	479	1049	8260
Non-Industrial (non-industrial location, e.g., chlorine release at a swimming pool)	258	171	4118
Poison in food or drink (including methanol)	222	2574	13500
Explosion and fire	212	924	5668
Deliberate/criminal incident	97	683	3223
Environmental contamination	84	111	7153
Carbon monoxide	66	55	1277
Transport incident	64	462	2679
Illegal drugs	56	50	3537
Medical product	27	45	1205
Mining accident	17	108	40
Consumer product	14	24	30
Self-harm	4	3	12
Unknown	1	4	24

In terms of the health effects of the most commonly identified chemicals, ‘unknown’ incidents (where the causative agent was not identified, reported or elucidated) accounted for 1421 deaths (23.7% of the total) and 11,563 injuries (22.8% of the total). Of the incidents where the causative agent was identified, methanol has caused the highest number of deaths and injuries at 2090 (24.9% of the total) and 9381 (18.5% of the total), respectively. As methanol was not the most common chemical agent, it highlights the severe health impacts methanol incidents can cause.

The next most common agents involved in incidents leading to high loss of life include petrochemicals (petrol/diesel/kerosene and liquefied petroleum gas) at 608 (10.1%), explosives (including munitions and fireworks) at 388 (6.5%) and chlorine at 122 (2.0%). When looking at the number of injuries caused, the agents involved are slightly different. Other than methanol and unknown agents, the next highest number of injuries from the detected chemical incidents involves benzene with 3425 injuries (6.4%), synthetic cannabinoids with 2943 injuries (5.8%) and ammonia with 2888 recorded injuries (5.7%).

### Methanol incidents

**Fig. 1:**
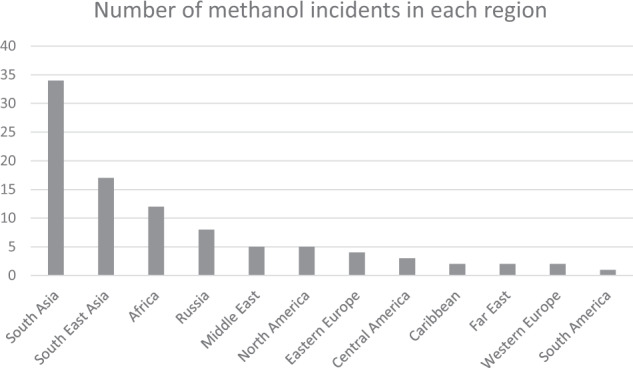
Number of methanol incidents detected through EBS, grouped by world region. This figure shows the distribution of the methanol incidents around the globe. This does not reflect the total number of these incidents, only those which were detected through EBS.

Methanol poisoning is responsible for most of the deaths and injuries detected through EBS. The problem is so widespread that it affects almost all the regions in the world; there is also a high mortality rate associated with these incidents as 92% of the detected incidents involve at least one or more deaths. The number of deaths and injuries tend to be higher in countries where alcohol is either not widely sold or is prohibitively expensive for most residents. While some of these are due to intentional ingestion of methanol, the majority of the detected incidents are due to outbreaks of people unknowingly consuming methanol-contaminated alcohol. Figure 1 shows the number of methanol outbreaks detected through EBS by world region.

Over half (54%) of reported methanol incidents occur in South and South East Asia, this could be for a variety of reasons including: poorer regulation on spirit production and distribution, reduced access to legitimate alcohol products, affordability of legitimate products versus bootlegged or locally manufactured alcohol, religious considerations (if the majority religion of the area prohibits consumption of alcohol, available products are likely to be unregulated and produced locally). Of course, it is not possible to draw definitive conclusions from this overview of the data, these are merely suggestions to try to explain the trend in methanol incidents.

Some recent methanol incidents have been related to the coronavirus pandemic, as it is believed by some that drinking alcohol offers protection from the virus. This has prompted an increased demand for alcohol that did not previously exist. In some countries where alcohol is more difficult to find, e.g., Iran, the demand has caused an increase in the availability of adulterated alcohol. In an ongoing incident, first detected in April, over 700 Iranians have died and more than 5000 have been injured due to methanol poisoning [19].

## Discussion

This paper has provided a description of the EBS activities undertaken, the main advantages and disadvantages of this strategy, examples of the data analysis carried out and some broad trends identified from this work. EBS was originally established to identify chemical incidents occurring in the EU that could be posted to RASCHEM, as required under the EU decision 1082/2013 when it came into force. Some of the incidents detected by this method were, or had the potential to be, cross-border incidents and were posted to RASCHEM and shared with the EU (Directorate-General for Health and Food Safety, DG SANTE). Performing regular EBS can be used to heighten situational awareness and be used to provide advice in the event of cross-border incidents to appropriate organisations and relevant agencies (e.g., EU SCHEER committee). The data have been used to contribute to awareness raising of chemical incidents through the creation of case studies and exercise scenarios, used in training courses, workshops and exercises on chemical incident preparedness and response. Reports on a specific region or a specific time period are also created, which identify trends in the type of agents involved in incidents, the health effects common to a region or caused by a particular agent. Under previous EU projects (e.g., ECHEMNET, EMETNET [15, 20]) a weekly digest of the recent events detected was sent out to an agreed mailing list, including project partners and key experts. These include experts from a variety of European countries, colleagues from Canada, Australia and from WHO and the European Commission.

This EBS method is not designed, and never was intended to, follow-up on the detected incidents. Aside from the fact that following-up on each incident would take a prohibitive amount of time, it was not in the scope of why EBS was initiated. While the original scope of EBS was to detect and report potential cross-border chemical incidents, with the aim of highlighting these issues for countries to take action if they deemed it appropriate, the scope was later broadened to include the rest of the world and WHO, so that they could liaise with the affected country if required. This increase in detected events allowed for the monitoring of certain broad trends, including the distribution of detected incidents by country/world region, the agents involved and the health burden (injuries/deaths) caused by these incidents. Trends in the types of incidents identified and chemical agents involved have also been found, highlighting methanol as one of the most commonly detected chemical incidents with the greatest health burden (deaths and injuries). In addition, details on which sources provide the most detected incidents, as well as the type of incident, have also been reported. The countries that were identified as having the highest number of detected incidents include USA, India, UK, China and Russia. The number of incidents detected in these countries is not necessarily a direct reflection of the number of incidents that occur but may reflect a difference in capacity for reporting incidents. As this methodology includes only cases with reported death and injuries, further work would be required to assess the public health impact and burden of disease associated with these events. In general, the USA and UK often report chemical incidents with fewer health effects whereas in India and China, the incidents that are detected tend to have more serious health effects (e.g., multiple deaths), while more minor incidents (e.g., with a small number of injuries) are generally less reported or detected through EBS.

A general observation is that while EBS tends to focus on picking up large-scale acute chemical incidents (such as explosions or major leaks at chemical industrial facilities) that may affect multiple countries, these actually account for a small proportion of the health effects recorded. The majority of the health burden is due to frequent, smaller-scale incidents, such as outbreaks of methanol poisoning. Further data analysis is required to better understand and provide evidence for any potential trends identified from the EBS data. For example, linking methanol incident data with existing data from national poison centres would validate information on the health effects of the incident (such as confirming number of persons affected, seriousness of exposure, required treatment and any follow-up that has been undertaken).

### Comparison with other methods/systems

Comparison of this overall EBS strategy with just one of the sources used (e.g., MediSYS) shows that the number of detected incidents using this EBS system is far higher (see Table 3 on sources of EBS information, MediSYS was responsible for only 3% of the events detected by this system in the last 12 months). A major advantage of this method is the number of sources used, which has been balanced for maximum detection of incidents against the time taken to search through sources (i.e., adding more sources would increase the time taken in performing EBS and would become prohibitive). Therefore, aligning this method with others would provide an even more comprehensive approach.

One limitation of this approach is that while there are many systems in place for communicable disease surveillance, there are comparatively few sites/tools available that focus on chemical incident and most of the sources used here are primarily designed for detection of biological incidents (e.g., outbreaks of communicable disease) [11]. As communicable disease surveillance is more established, it makes sense to utilise as much as possible the tools, resources and strategies that are currently available via communicable disease surveillance, rather than try to establish separate tools for chemical incident surveillance from scratch.

An example of where chemical and communicable disease surveillance can overlap is through syndromic surveillance, which searches for symptoms exhibited by exposed individuals, rather than confirmed incidents. While this is primarily used for communicable disease surveillance, adding symptoms of chemical exposure to the list of symptoms of communicable diseases is a straightforward way of enabling the detection of chemical exposures as well. Some European Poison Centres have specific syndromic surveillance for known chemicals of concern, enhancing this process for chemical incidents [21]. This method could also allow the detection of exposures/outbreaks of unknown aetiology, which otherwise may not be detected as chemical incidents. It could also improve the follow-up and monitoring of emerging incidents, by identifying any additional affected individuals.

Greater integration with other sources of data would allow a more unified, comprehensive surveillance system as described in the JEE Tool [22] and IHR to detect, notify, respond and follow-up (recovery/biomonitoring) a wider range of chemical incidents (not just acute chemical incidents, which are the focus of this work, but including, e.g., chronic and environmental exposures). This could be further integrated with surveillance systems for other health threats, to compile a comprehensive all hazards approach to current health threats in a given population.

The EBS process outlined in this paper has identified some trends (as well as caveats and limitations), such as the locations where more incidents are detected, the different health burden of the agents causing the detected incidents, the identification of methanol incidents as having the most significant health burden (even when it is not the most common causative agent in the detected incidents) and differences in the sources of incidents that have been used. The limitations of this method include: the details from the reports may be unreliable and the difficulty in verifying or confirming information from the initial detected reports. While it is possible to search for updates on specific incidents, e.g., for the most recent/accurate casualty information, it is not feasible to do this routinely, but can be a useful tool to increase awareness of the type and range of events, and alert public health agencies to potential cross-border health incidents. One way to address this would be to link with, e.g., hospital data to provide reliable figures on those injured from chemical incidents. However, this is not really in the scope of EBS, a system that is designed to detect incidents soon after they have occurred, to enable the early warning and notification of others involved in the response to such incidents.

More could to be done to improve the sustainability of this type of surveillance activity, both at a national and international level, e.g., across the EU. For example, more in-depth data analysis, follow-up of chemical incidents to gather further information and track recovery from these incidents, notifying/alerting relevant stakeholders on a more routine basis (currently it is done ad hoc) and linking the data with other surveillance systems or existing databases of chemical incidents. An additional improvement would be the increased automation of the system, as it requires a significant number of person-hours to routinely look through the sources, evaluate each incident, cross-check the information and manually enter incidents into the database. Google Alerts and automated updates from other sources used (e.g., RSOE EDIS) already detect potentially relevant incidents but this would need to be applied to all sources, to reduce time spent on manually scanning and detecting incidents. There is also the possibility of linking with other surveillance systems nationally and internationally. At first this could include other surveillance systems for chemical incidents, but could be incorporated into an all-hazard surveillance approach.

Previously there was a network in place for utilising the incidents detected through EBS, as under the ECHEMNET project, incidents were forwarded to network colleagues in EU countries, Poison Centres and posted to RASCHEM if there was potential for the incident to become cross-border [15]. As the project came to a close and the network is no longer active, it would be beneficial to explore alternative sustainable mechanisms to continue this work. EU projects and Joint Actions provide valuable mechanisms to develop and enhance chemical detection and response arrangements [23]. EBS was initiated to support/enhance arrangement for the detection and response to cross-border health threats in Europe but has been found to be a valuable repository of information of international events. This paper has described the methodology used to undertake EBS and demonstrates how this has been communicated to established public health networks and used to strengthen public health arrangements. Finding sustainable mechanisms to maintain and enhance EBS continues and these activities could support strengthening of chemical incident preparedness and reduce the global health effects from chemicals.
